# Enhanced Clinical Decisions for Management of Benign Prostatic Hyperplasia using Patient-Reported Outcomes: Protocol for a Prospective Observational Study

**DOI:** 10.21203/rs.3.rs-4308293/v1

**Published:** 2024-05-06

**Authors:** Alexander P. Glaser, Abigail R. Smith, Dacey Maglaque, Brian T. Helfand, Rowida Mohamed, Hosanna An, Melissa Marquez, Pooja Talaty, Padraig Carolan, Aaron M. Geller, Francesca R. Farina, Sally E. Jensen, James W. Griffith

**Affiliations:** NorthShore University HealthSystem; Northwestern University; NorthShore University HealthSystem; NorthShore University HealthSystem; University of Chicago; University of Chicago; University of Chicago; NorthShore University HealthSystem; Northwestern University; Northwestern University; University of Chicago; Northwestern University; University of Chicago

**Keywords:** Lower urinary tract symptoms, Prostatic Hyperplasia, Patient Reported Outcome Measures, Symptom Assessment, Qualitative Research

## Abstract

**Background:**

Lower urinary tract symptoms (LUTS) due to benign prostatic hyperplasia (BPH) significantly impact quality of life among older men. Despite the prevalent use of the American Urological Association Symptom Index (AUA-SI) for BPH, this measure overlooks key symptoms such as pain and incontinence, underscoring the need for more comprehensive patient-reported outcome (PRO) tools. This study aims to integrate enhanced PROs into routine clinical practice to better capture the spectrum of LUTS, thereby improving clinical outcomes and patient care.

**Methods:**

This prospective observational study will recruit men with LUTS secondary to BPH aged ≥ 50 years from urology clinics. Participants will be stratified into medical and surgical management groups, with PRO assessments scheduled at regular intervals to monitor LUTS and other health outcomes. The study will employ the LURN Symptom Index (SI)-29 alongside the traditional AUA-SI and other non-urologic PROs to evaluate a broad range of symptoms. Data on comorbidities, symptom severity, and treatment efficacy will be collected through a combination of electronic health records and PROs. Analyses will focus on the predictive power of these tools in relation to symptom trajectories and treatment responses. Aims are to: (1) integrate routine clinical tests with PRO assessment to enhance screening, diagnosis, and management of patients with BPH; (2) examine psychometric properties of the LURN SIs, including test-retest reliability and establishment of clinically meaningful differences; and (3) create care-coordination recommendations to facilitate management of persistent symptoms and common comorbidities measured by PROs.

**Discussion:**

By employing comprehensive PRO measures, this study expects to refine symptom assessment and enhance treatment monitoring, potentially leading to improved personalized care strategies. The integration of these tools into clinical settings could revolutionize the management of LUTS/BPH by providing more nuanced insights into patient experiences and outcomes. The findings could have significant implications for clinical practices, potentially leading to updates in clinical guidelines and better health management strategies for men with LUTS/BPH.

**Trial registration:**

This study is registered in ClinicalTrials.gov (NCT05898932).

## INTRODUCTION

Lower urinary tract symptoms (LUTS) attributable to benign prostatic hyperplasia (BPH) are common, affecting approximately 33% of Medicare beneficiaries.[1] As the most common condition contributing to non-neurogenic LUTS in men, BPH is also the focus of frequently updated medical and surgical management guidelines that directly influence provider decision-making and patient care.[2, 3] The burden of LUTS/BPH is expected to increase, both in the US and worldwide, due to shifting demographics and a static urologic workforce.[4] To meet this demand, there is an urgent need to develop and refine efficient strategies to assess, triage, and enhance the care of patients through standardized outcome measures and optimized care coordination, including remote assessment.

Measuring outcomes provides a way to track progress, adjust treatments, monitor quality of care, and help patients achieve optimal outcomes. For decades, the American Urological Association Symptom Index (AUA-SI) has been the patient-reported outcome (PRO) measure used in men with BPH, as recommended in the “Surgical Management” guidelines updated as recently as 2023.[2, 5] The AUA-SI, however, has several limitations that leave room for improvement. First, the AUA-SI omits urinary incontinence and pain, which in the Symptoms of Lower Urinary Tract Dysfunction Research Network (LURN) cohort, were present in 51% and 17% of treatment-seeking men, respectively, despite exclusion of men with infection or urinary pain as a primary complaint.[6, 7] Urgency urinary incontinence, stress urinary incontinence, and post-void dribbling were all associated with higher LURN-SI scores, AUA-SI scores, and bother, and thus mandate assessment.[7, 8] Furthermore, a proportion of men present with pain, which can be an important differential diagnostic marker (e.g., between BPH and chronic prostatitis/chronic pelvic pain syndrome). Pain may also currently be underassessed in the postoperative setting.[9] Second, the AUA-SI yields a single score for overall LUTS, which is laudable for its simplicity and may facilitate communication with patients. However, a single score may be insufficient when symptoms are differentially impacted by treatment or have different relationships with biomarkers, diagnostic subgroups, and other clinically important variables. Third, the methodology used to develop the AUA-SI was specific to BPH, which is advantageous once the patient has been properly diagnosed, but patients presenting to the urology clinic with LUTS may have other diagnoses such as kidney stones, infection, chronic prostatitis, pelvic floor dysfunction, and stricture disease. The AUA-SI, although designed for BPH, has been used in a variety of conditions it was not designed for, including prostate cancer and overactive bladder syndrome.[10, 11]

With an awareness of the need to create an updated outcome tool in urology, LURN created new questionnaires in urology including the 10- and 29-item LURN Symptom Indices (SIs).[12, 13] The LURN Symptom Index SI-29 was designed as a comprehensive tool, whereas the one-page LURN SI-10 can be extracted from the broader LURN SI-29 and was designed for clinical use analogous to the AUA-SI. Both the LURN SI-29 and SI-10 were developed as PROs for patients with LUTS, using both qualitative and quantitative methods, and have shown initial validity evidence in people with LUTS.[8, 12–15] What is needed now is research on specific conditions, such as rigorous validation of the psychometric properties of these questionnaires in LUTS/BPH. In order for these symptom questionnaires to improve outcomes, they must be connected to actionable phenomena in the clinic (e.g., providing the patients with tailored information, treatment, and/or referrals based on their scores). Toward this end, the present study will administer PROs about both urologic and non-urologic symptoms into the routine care of patients with LUTS/BPH.

### Study Objectives

#### Aim 1: Integrate routine clinical tests with PRO assessment to enhance screening, diagnosis, and management of patients with BPH.

PROs provide a cost-effective assessment strategy that can improve patient care by allowing for frequent surveillance along with routine objective clinical variables (e.g., post-void residual, prostate volume, intravesical prostatic protrusion), will provide unique predictive power for LUTS severity over time. Understanding a broad set of health factors, such as sleep disturbance and depression, which may exacerbate LUTS, will facilitate counseling and interventions to improve in-clinic and remote evaluation and treatment.[16]

#### Aim 2: To examine psychometric properties of the LURN SIs, including clinically meaningful differences in men with BPH receiving known effective treatment.

Longitudinal data will enable calculation of effect sizes of the LURN SI-10 and SI-29 from baseline to follow-up in the context of usual care, including guideline-driven treatment of BPH. LURN SIs will be anchored to patients’ ratings of global change (PGI-I), and longitudinal assessments will provide quantitative estimates of minimally important differences (MIDs).[17, 18] We hypothesize that MIDs will be approximately 0.5 SDs. Repeated assessment on a subset of patients will allow estimation of test-retest reliability.

#### Aim 3: Create care-coordination recommendations to facilitate the management of persistent symptoms and common comorbidities measured by PROs.

PROs provide a patient-centered way to assess problems that may persist after treatment has been initiated. For example, surgical treatments can lead to transient but distressing symptoms, including pain, urgency, and/or incontinence, which require monitoring after surgery.[9] Both LUTS and comorbid symptoms cause bother for the patient and reduce the quality of life, so it is important to monitor these symptoms and coordinate care so that these symptoms are effectively managed.[6, 15] We will use qualitative interviews with patients and providers to inform the creation of a care-coordination recommendations that can be implemented for patients with BPH. Patients and providers will be interviewed about unmet needs in between clinic visits to develop better ways to follow these symptoms using a combination of in-clinic visits, telehealth, asynchronous contacts, and remote symptom monitoring. We hypothesize that patient and stakeholder input will suggest care-coordination recommendations that have the potential to increase quality of care, and in turn support better outcomes for patients with BPH.

## METHODS

### Ethical approval

This study was approved by the Institutional Review Board of Northwestern University (IRB STU00217126), which served as the single IRB of record. This study is registered in ClinicalTrials.gov (NCT05898932).

### Study design, setting, and population

#### Aims 1 and 2:

Men with BPH aged 50 years and older will be recruited from urology clinics at NorthShore University HealthSystem (“NorthShore”). Recruited patients with BPH will undergo standard of care treatment as determined by their urologist. To obtain a sample that is representative of BPH patients, as well as to generalize to patients receiving different kinds of treatment, the sample will be stratified by whether they are to receive medical/non-surgical management (n = 200) or procedural/surgical management (n = 100). Participants will be allowed to crossover between treatment arms if their clinical treatment type changes as determined by shared decision making with their urologist (e.g., if a participant in the medical management arm decides to undergo surgery during routine clinical care).

Both new and returning patients undergoing medical or surgical treatment for BPH will be eligible. Eligible patients (see Table 1 for inclusion and exclusion criteria) will be identified in two ways: (1) a research coordinator will screen the electronic health record (EHR) for upcoming clinic appointments to identify patients who may be eligible. A coordinator will contact these patients by phone to describe the study and obtain informed consent remotely, which will be documented using the Research Electronic Data Capture System (REDCap). Alternatively, (2) eligible patients who present for a clinic visit (either in-person or telehealth) will be referred to the coordinator to discuss the study, obtain informed consent (again either in-person or remotely), and document the consent and participant details in REDCap. During these interactions, research staff will explain the contents of the consent form, focusing on the purpose of the study, study procedures, study duration, participants’ rights and responsibilities, potential risks and benefits, and the use of participant’s protected health information for research purposes. Participants will be given the opportunity to ask questions about the study

2. Positive urine culture

and will be provided with as much time as needed to review information about the study to reach a voluntary decision about participation.

#### Aim 3:

The study will encompass a diverse group of healthcare professionals responsible for the care of individuals with LUTS/BPH, including urologists, physician assistants, and nurses, as well as the patients themselves who are coping with BPH.[19] This method seeks to include a wide array of backgrounds within the participant pool, thus enriching the study’s insights by capturing the full spectrum of experiences related to BPH.

### Data collection

All participants will complete a baseline visit after consent where demographic and clinical information will be collected. Routinely collected objective clinical data from the EHR will be analyzed in combination with PROs. In our protocol, we included health questionnaires specific to the management of LUTS/BPH, listed in Table 2. Participants in the medical arm will complete questionnaires at baseline and every 4 weeks for 24 weeks (Table 2.1); participants in the surgical arm will complete questionnaires at baseline, pre-op, and Weeks 1, 2, 4, 6, 8, 12, and 24 post-surgery (Table 2.2). Patients undergoing surgery will have more frequent queries in the immediate postoperative period (Table 2.2), as many men report bothersome symptoms of frequency, urgency, pain, and/or incontinence in this timeframe; thus, this period, in particular is a target for better characterization and management of symptoms. The use of PROs is already part of the management guidelines for BPH; the overarching hypothesis of this project is that a brief (i.e., clinically feasible) set of questions can aid in the management of BPH and can improve upon the existing method of the AUA-SI. We recognize that the proposed series of measures is longer than one might use in clinical practice, but a comprehensive assembly of questionnaires is necessary for research purposes to clarify which measures would need to be dropped versus retained for optimal use in the real-world clinic. Data analyses will also include the LURN SI-10 score, which is a summary score that can be extracted from the LURN SI-29.

For assessment of test-retest reliability, patients will receive the LURN SI-29 and AUA-SI questionnaires in two instances, approximately 2 weeks apart. We will target 2 weeks between each questionnaire administration but will allow a 1–3-week time interval between administrations. When feasible, patients who provide consent prior to their baseline visit will complete the questionnaires 2 weeks (range 1–3 weeks) prior to baseline and then again at the baseline visit. Patients who provide consent at the baseline visit and have a scheduled diagnostic procedure at least 1 week but no longer than 3 weeks later (e.g., diagnostic cystoscopy and/or prostate transrectal ultrasound), with no treatments or interventions planned in the interim, will complete questionnaires at the baseline clinic visit and again 2 weeks later (range 1–3 weeks). The questionnaires will be delivered via Epic MyChart, if available, or alternately using REDCap, on paper, or if needed over the telephone (e.g., in patients with insufficient vision). A research coordinator will contact participants to remind them of these questionnaires in order to minimize missing data. Mode of administration will be recorded.

To assess MIDs, we will administer the Patient Global Impression of Improvement (PGI-I) as a measure of global change at each post-baseline time point. The PGI-I is a single-item questionnaire that asks respondents to rate their condition compared with prior to treatment on a 7-point scale from very much worse (1) to very much better.[17] LURN SI-29 and SI-10 scores at 12 weeks post-baseline will be used to assess changes in scores since baseline. At the end of the study, participants will be invited to complete a patient-response burden questionnaire to provide a better understanding of how the participants perceive the RROs,

#### Qualitative Data Collection

Participants in qualitative interviews will include patients and health care providers. To maximize convenience, interviews will be conducted via telephone or secure virtual interview (e.g., Zoom) by trained interviewers. Health professionals will be interviewed once; patients will be interviewed at three time points: baseline, post-treatment, and follow-up. The first two interviews with patients as well as the ones with health care professionals will inform Version 1 of the care coordination recommendations. Subsequently, the third patient interview will focus on the refinement of the care coordination recommendations into Version 2 based on participants’ feedback. Participants will provide informed consent prior to each interview and will be asked to verbally confirm their consent at the beginning of the interviews. Trained researchers from Northwestern University and the University of Chicago will use semi-structured qualitative interview guides focusing on how PROs can contribute to decision-making and help patients address unmet needs and health care coordination gaps. Interviews will be audio-recorded and transcribed. Each interview is expected to be less than 20 and 60 minutes for health care professionals and patients, respectively. The interviewing process will continue until no new themes emerge, with no more than 20 interviews with health professionals and 40 interviews with patients. Health care professionals will receive $100 for each interview; patients will receive $40 for each interview, resulting in a total compensation of $120 for patients who complete all three interviews.

### Analysis

Patient demographics and characteristics will be described using frequencies and percentages for categorical variables. Continuous data will be summarized using means, standard deviations (SDs), and as appropriate medians and interquartile ranges for variables with non-Gaussian distributions. The distribution of treatments received, and duration of medication treatment will also be assessed using similar statistics. Longitudinal trajectories of questionnaire responses will be explored using spaghetti plots and other graphical strategies.

#### Aim 1: Identify health variables from the PROMIS Profile that predict trajectories of LUTS within the individual patient.

The primary hypothesis of Aim 1 is that health variables from routine care combined with PROs on health domains relevant to BPH (e.g., sleep disturbance, depression) will predict changes in LUTS over time in this 6-month study. We will assess these associations with LUTS over time using linear mixed models with random participant intercepts and slopes. Separate models will be run for each urinary outcome of interest (SI-29 subscales: incontinence, pain, voiding, urgency, nocturia, as well as the SI-29 total score and SI-10 score). Covariates of interest will include objective clinical measures as well as PROMIS measures (e.g., sleep disturbance, depression, pain) and the type of treatment received. Time-dependent linear mixed models will allow exploration of longitudinal mediation. For example, we expect obesity and sleep apnea will lead to sleep disturbance, which in turn affects LUTS. In these models, LUTS is a dependent variable regressed on sleep disturbance at the previous time point, which in turn is regressed on the participants obesity and sleep apnea status.

We will also explore other approaches to connect patient phenotype to LUTS trajectory. We will create subgroups based on variables such as treatment received, clinical cut points of PROMIS measures, and objective clinical parameters (e.g., obese vs. non-obese). We will assess changes in SI’s from baseline to 12- and 24-weeks and compare these differences across different subgroups. We will further explore latent class trajectory models to create homogenous groups of participants based on their longitudinal urologic symptoms as measured by the SI-10 and SI-29 over follow-up. This statistical method theorizes that a participant’s urologic trajectory is driven by their latent class membership. The “latent class” in these models is not directly observed but rather inferred from patterns in the data. The optimal latent class solution will be identified using the Bayesian Information Criterion (BIC) fit statistic (lower BIC indicates a better fit). Once these latent classes are identified, multinomial logistic regression will be used to predict group membership using baseline variables, including objective clinical measures and questionnaire scores. Treatment patterns in each group will also be explored descriptively.

#### Aim 2: Examine psychometric properties of the LURN SIs, including clinically meaningful differences in men with BPH receiving known effective treatment.

##### Test-retest reliability

The premise of test-retest reliability is that if a construct (e.g., urinary urgency) does not change, then the questionnaire score should also not substantially change. Thus, changes in scores without intervention can be used as an index of measurement error. LUTS are known to vary over short timeframes; therefore, any changes in questionnaire responses could be due to true changes, not measurement error. We expect, however, the test-retest reliability to be consistent with other LUTS questionnaires.

Test-retest reliability will be assessed for both the LURN SIs and the AUA-SI in two ways. First, we will use an intraclass correlation coefficient (ICC) from a two-way mixed-effects model using absolute agreement assumptions. This model is appropriate for scores obtained multiple times from the same patients, and the absolute agreement measure includes any systematic differences in scores across the two instances (i.e., differences in mean scores) as relevant to the calculation. Given our expected sample size, an ICC estimate above 0.9 would indicate excellent reliability, estimates greater than 0.75 or 0.5 would indicate good or moderate reliability, respectively.[31]

Second, we will calculate the coefficient of reliability (CR), an absolute measure of test-retest reliability that quantifies measurement error. The CR is an absolute measure in the sense that it assesses absolute differences (as opposed to consistency, which does not guarantee agreement to be high) and represents the maximum difference between two measurements with 95% probability. It has the advantage of being measured in the same units as the questionnaire, facilitating clinical interpretation. The Pearson correlation will also be calculated as necessary to make comparisons of test-retest reliability with other published questionnaires. The two pre-treatment visits will be the basis for an ICC for both measures. We will also examine graphically the consistency of the LURN SIs over the two-week test-retest period.

##### Minimally Important Differences (MIDs)

MIDs are an important psychometric property to establish because statistical differences in PRO measures do not always translate to meaningful differences in patient perception or result in changes in patient management.[32] MIDs should be based on a variety of methods that converge (i.e., triangulation). Three commonly used methods are distribution methods, anchor-based methods, and opinion-based methods; although no single method has been established as preferred, a combination of methods ensures that robust and generalizable MIDs are established. We will use an anchor-based method as primary, with distribution-based and opinion-based methods as secondary/sensitivity analyses to validate our results.

We will administer the PGI-I at each post-baseline time point as the anchor questionnaire. The 7-point PGI-I will be collapsed into three levels (Better, No Change, and Worse), as it is unlikely that we will have sufficient endorsement at all seven ratings for analysis. We will then describe the mean and standard error (SE) of change scores at each of the three levels and compare mean differences across the three ratings of improvement. We hypothesize that mean differences in LURN SI-10 scores and LURN SI-29 subscale scores will be significantly different for patients rating their improvement as Better, No Change, and Worse on the PGI-I based on parametric or non-parametric Analysis of Variance (ANOVA), as appropriate. Inspection of the change difference, i.e., the difference in mean subscale score changes between the better and the average of the No-Change and Worse ratings, will guide the determination of Preliminary MIDs for each subscale score. We will then verify our preliminary MIDs by using them to assess their sensitivity and specificity for predicting a response of “Better” using Receiver Operating Characteristic (ROC) Curves.

We will then examine the distributions of the LURN SI-10 scores and SI-29 subscales before and after treatment (i.e., baseline vs. 12-week follow-up), as well as the distributions of difference scores. Multiples of the SD of the change scores (e.g., 0.2, 0.5) will be calculated and compared with the preliminary MIDs identified by the anchor approach. We expect that the MIDs identified by the anchor approach will correspond to approximately 0.5 SDs in the change score, representing a meaningful change for many health-related quality of life instruments.[33, 34] MIDs identified by both methods will be shared with stakeholders (i.e., providers who see patients with BPH; patients with BPH) to ensure that these values are clinically sensible. We will also examine changes in the LURN SIs in relationship to the established clinically meaningful change (3 points or more) on the AUA-SI.[17] The final recommended MIDs will be determined by synthesizing these various methods.

##### Qualitative data analysis

Qualitative interviews with patients and providers will be transcribed and imported into the qualitative analysis software Depose.[35] Two coders will review a sample of five transcripts to create an initial codebook. They will then meet to discuss and revise the coding scheme. The two coders will independently recode the same transcripts using the updated coding scheme, followed by a second meeting to further refine existing codes and add new ones. Subsequent transcripts will be coded separately by the two coders, who will have regular meetings to maintain consistency. Regular meetings with the research team at large will inform the coding process and resolve any coding discrepancies. The codebook will represent themes expressed by the participants across the interviews. Themes, concerns, and symptoms from the qualitative interviews will be used to determine prevalent topics for inclusion in care coordination. We will then organize the themes around clinical problems that could be resolved by concrete action (e.g., referral to sleep medicine for insomnia, referral to psychology/psychiatry for anxiety).

##### Sample size and power considerations

Based on prior publications, we expect – under usual treatment – approximately 70%, 20%, and 10% of men to report Better, No change, and Worse, respectively, on the PGI-I at the 12-week assessment.[36] Assuming a sample size of 300 from Aim 1, this represents 210, 60, and 30 participants, respectively. Using a conservative Type I error rate of alpha = 0.01 to account for pairwise testing across the three groups, we estimate that we will have at least 80% power to detect effect sizes as small as *d* = 0.5 (a medium-sized effect)[37] comparing the better and no-change groups. We would also have 80% power for effects as small as *d* = 0.7 comparing the better and worse groups, and as small as *d* = 0.8 comparing the no-change and worse groups. Although some of these effects are large in size (i.e., *d* = 0.8), prior work using the PGI-I to assess changes in the AUA-SI in four placebo-controlled randomized controlled trials found effect sizes of 0.75–1.75 between the three groups.[17] Therefore, our study will be sufficiently powered with 300 participants assuming similar magnitude of change in the LURN SIs. For test-re-test reliability we calculated that 60 patients will be needed to obtain an ICC estimate of 0.8 with a 95% confidence interval half-width of no more than 0.1; therefore, participants will be approached until 60 patients have completed two scorable questionnaires.[38]

##### Data monitoring and quality control

The Safety Monitor of the study will provide oversight for data and safety monitoring for this study. The Safety Monitor and the study teams will meet bi-annually to review the data and any adverse events.

## Discussion

The goal of this project is to use newly developed LURN SIs to improve the clinical care of patients with LUTS/BPH. The integration of new patient-centered tools will improve evaluation and clinical decision-making by including symptoms not commonly measured in men using standard questionnaires, such as urinary incontinence. Brief PROs will allow for frequent monitoring of LUTS through remote assessment. Using care-coordination recommendations, the health care team can be more responsive to post-treatment symptom changes, resulting in reduced bother from LUTS and higher quality of life in patients with BPH.

Strengths of this study include its longitudinal design, questionnaires that capture symptoms relevant to BPH, integration of questionnaires with data from the medical record, and mixed methods comprising quantitative and qualitative methods. We have taken care to accommodate various preferences by allowing patients to provide PROs through multiple means, including EPIC MyChart, traditional paper-and-pen surveys, or phone interviews. This approach ensures that individuals with varying levels of technological proficiency can participate, minimizing the risk of missing data and bolstering the overall representativeness and reliability of our study’s findings. Several limitations are important to note. Patient recruitment for our study is primarily conducted within a single health care organization (NorthShore), which could limit the generalizability of our results. However, analysis and qualitative work is spread across three major academic institutions in Chicago (NorthShore, Northwestern University, and the University of Chicago). Variations in practice patterns, health care staff expertise, and patient perspectives across different settings, institutions, or regions may impact the applicability of our findings to other contexts. A potential limitation of our study is self-selection bias, wherein our sample is composed of participants who choose to respond to our recruitment efforts. This could introduce systematic differences between participants and non-participants, potentially affecting the generalizability of our findings. Another limitation is the observational rather than experimental nature of the study. This was an intentional choice as our goal is to build toward a randomized controlled trial. Lastly, we must address potential biases related to self-reported data, including social desirability bias and memory recall issues. Respondents may provide answers that they perceive as socially acceptable or desirable, which may not reflect their true thoughts or behaviors. Moreover, memory recall issues could introduce errors or inaccuracies into our data. We will be vigilant in considering these potential biases throughout our analysis and interpretation of the results.

In the future, we hope to incorporate the findings of this study, including the use of the LURN SIs, into the clinical practice guidelines for patients with BPH. Future plans may also include randomized-controlled trials on potential drivers of LUTS identified in this study. For example, a clinical test of Aim 3 would be a trial of whether implementation of the care coordination recommendations results in better outcomes for men with BPH and/or decreased healthcare utilization in these men. Results of this study may also inform the development of patient self-management tools. Finally, although this project focuses on the population of patients with LUTS secondary to BPH, the LURN SIs can be implemented in a variety of disease states, including stricture disease, pelvic floor dysfunction, and prostate cancer, which creates additional avenues for research.

## Conclusion

The primary goal of PROs is to give the patient a voice in their health care by having them report on perceived symptoms, bother, and impact. PROs have already had a long history of success with the AUA-SI in clinical care and research. However, after decades of the AUA-SI, it is time for some innovative changes and improvements that more broadly capture the symptoms and needs of men with LUTS/BPH, and to directly connect them to comorbidities and QOL. Implementation of this study will ensure that the LURN SIs do more than merely collect data, but rather use them to guide treatment, thereby improving the care of men with BPH.

## Figures and Tables

**Table 1:** Inclusion and exclusion criteria

Inclusion Criteria	Exclusion Criteria
1) Male sex	1) Female sex or intersex
2) Age 50 years or older	2) Younger than 50 years of age
3) Diagnosed by physician with BPH	3) Being a prisoner or detainee
4) Able and willing to complete questionnaires	4) Gross hematuria
5) Able and willing to provide informed consent	5) Interstitial cystitis
6) Ability to read, write, and speak in English	6) Pelvic or endoscopic genitourinary surgery within the preceding 6 months (not including diagnostic cystoscopy)
7) No plans to move from study area in next 6 months	7) History of cystitis caused by tuberculosis, radiation therspy, or Cytoxan/cyclophosphamide therapy
	8) Ongoing symptomatic urethral stricture
	9) Current chemotherapy or other cancer therapy
	10) History of lower urinary tract or pelvic malignancy
	11) Severe neurological of psychiatric disorder that would prevent study participation (e.g., bipolar disorder, psychotic disorder, Alzheimer’s Disease)
	12) Current moderate or severe substance use disorder
**Deferral Criteria**	
1) Microscopic hematuria without appropriate workup per AUA/SUFU Guidelines	
2) Positive urine culture	

**Table 2.1:** Study questionnaires, medical/non-surgical intervention (Schedule of assessments: baseline, inter-visit follow-up, and clinic assessments)

	# Items	Week-2	Baseline (clinic)	Week 4	Week 8	Week 12 (clinic)	Week 16	Week 20	Week 24 (clinic)
**LURN SI-29** [12]	28								
**PROMIS–29 Profile v2.1** [20]	29								
**AUA-SI** [5]	8								
**PGI-I** [21]	1								
**National Cancer Institute Quick Food Scan** [22]	17								
**3-Day Voiding Diary** [23, 24]	9								
**PROMIS^®^ Squale v1.0 – Gastrointestinal Constipation 9a** [25]	9								
**PROMIS^®^ Scale v1.0 – Gastrointestinal Diarrhea 6a** [25]	6								
**SHIM** [26]	5								
**MSHQ-Ed** [27]	4								
**SBQ** [28]	8								
**PROMIS^®^ Item Bank v2.0 – Cognitive Function-Short Form 4a** [29]	4								
Patient Feedback Survey (optional)* [30]	6								

Note: Study time points (e.g., Week 4, Week 8) are approximate – Participants will be given a time window of ± 2 weeks. The minimum time between assessments will be approximately 4 weeks. Time windows may be adjusted based on COVID-19 protocols (e.g., if a clinic visit is converted to telehealth).

* Adapted from Atkinson et. al.

**Abbreviations:** LURN SI-29: LURN SYMPTOM INDEX-29; PROMIS: Patient-Reported Outcomes Measurement Information System; AUA-SI: The American Urological Association Symptom Index; PGI-I: Patient Global Impression of Improvement; SHIM: Sexual Health Inventory for Men; MSHQ-Ed: Male Sexual Health Questionnaire-Ejaculatory Dysfunction; SBQ: STOP-BANG questionnaire

**Table 2.2:** Study questionnaires, surgical/procedural intervention (Schedule of assessments: baseline, inter-visit follow-up, and clinic assessments)

	# Items	Baseline (clinic)	Preop**	Week 1	Week 2	Week 4 (clinic)	Week 6	Week 8	Week 12 (clinic)	Week 24 (clinic)
**LURN SI-29** [12]	28									
**PROMIS–29 Profile v2.1** [20]	29									
**AUA-SI** [5]	8									
**PGI-I** [21]	1									
**National Cancer Institute Quick Food Scan** [22]	17									
**3-Day Voiding Diary** [23, 24]	9									
**PROMIS® Squale v1.0 – Gastrointestinal Constipation 9a** [25]	9									
**PROMIS® Scale v1.0 – Gastrointestinal Diarrhea 6a** [25]	6									
**SHIM** [26]	5									
**MSHQ-Ed** [27]	4									
**SBQ** [28]	8									
**PROMIS® Item Bank v2.0 – Cognitive Function- Short Form 4a** [29]	4									
Patient Feedback Survey (optional)* [30]	6									

Note: Study time points (e.g., Week 4, Week 8) are approximate – Participants will be given a time window of ± 2 weeks. The minimum time between assessments will be approximately 4 weeks. Time windows may be adjusted based on COVID-19 protocols (e.g., if a clinic visit is converted to telehealth).

* Adapted from Atkinson et. al.

** If surgery is not immediately scheduled, complete questionnaires every 4 weeks until surgery is scheduled

**Abbreviations:** LURN SI-29: LURN SYMPTOM INDEX-29; PROMIS: Patient-Reported Outcomes Measurement Information System; AUA-SI: The American Urological Association Symptom Index; PGI-I: Patient Global Impression of Improvement; SHIM: Sexual Health Inventory for Men; MSHQ-Ed: Male Sexual Health Questionnaire-Ejaculatory Dysfunction; SBQ: STOP-BANG questionnaire

## Abbreviations

AUA-SI: American Urological Association Symptom Index
BPH: benign prostatic hyperplasia
LUTS: lower urinary tract symptoms
LURN: Symptoms of Lower Urinary Tract Dysfunction Research Network
LURN SI-29: LURN Symptom Index 29
LURN SI-10: LURN Symptom Index 10
MSHQ-Ed: Male Sexual Health Questionnaire-Ejaculatory Dysfunction
PGI-I: Patient Global Impression of Improvement
PRO: patient-reported outcome
PROMIS: Patient-Reported Outcomes Measurement Information System
SBQ: STOP-BANG questionnaire
SHIM: Sexual Health Inventory for Men
UI: urgency urinary incontinence
SD: standard deviation
